# Annotations

**Published:** 1900-03-24

**Authors:** 


### Annotations.

### Open-Air Treatment and its Teachings.

The continued influx of reports from all parts, many of them places the climates of which have the reputation of being far from salubrious, as to the benefits derived by phthisical patients from sleeping in the open air, raise very serious questions about the ordinary habits of nine-tenths of civilised human beings. It may be very difficult to ensure a full and complete ventilation of the rooms in which we live and work and have our daily being; we cannot consider ourselves alone, and unless all are provided with suitable clothing it clearly would be impossible in cold weather to permit of free currents of icy wind which people clad for arctic weather would enjoy. Nay, we will go further and admit that such arctic clothing as would be necessary for an open-window life on some days in mid-winter would, to a large extent, be incompatible with many avocations. But at night each man is his own master. For seven or eight hours out of the 24 most people can decide whether they will breathe pure air or foul, and judging from the position of the window-sashes as we sometimes see them while walking through the streets in the early morning, even in summer, we should say that the vast majority deliberately choose the foul air to the pure. How much ill-health is caused by this deliberate exclusion of fresh air during the hours of sleep it is impossible to say, but without doubt it is a very large amount. The idea that a room can be efficiently ventilated through such a mere chink as is afforded by lowering the sash about an inch is mere nonsense. Unless there is a fire in the room nothing short of a widely open window can afford enough ventilation to make the air inside a room equal or indeed at all approach the air of the street in purity. A very simple experiment with a tobacco pipe or with any strong scent will serve to show how long it takes to renew the air of an ordinary room, and for how long any impurity in the air of such a room must enter time after time into the lungs of those who sleep there. Perhaps one result of the war and of the return to England of a large number of men who have had practical experience of the advantage of sleeping in the open, may be the dissipation of the prevailing belief in the deleterious nature of night air. Perhaps; but we doubt it. Nothing is more engrained in the multitude than the love of stuffiness.

### The Busy Physician's Public Work.

To those on whose hands time hangs heavily, and whose pockets are but lightly filled with cash, the lot of the consultant in full practice appears worthy of much envy, for guineas are thought to be a full reward for any quantity of work. Such people, however, perhaps but feebly realise how hard the work sometimes is, or how often even the guineas have to be pushed aside in deference to the self-imposed but unremunerative duties which accumulate upon those whose names have once been brought prominently before the public eye. The mere correspondence of a physician in full practice is in itself an exhausting task, and no sooner does such a man show an interest in any philanthropic work, especially if he should happen to possess the power of making happy little speeches, than he is overwhelmed with requests to attend meetings, take chairs, propose resolutions, and, worse than all, to eat dinners innumerable. Public meetings, like all appointments for fixed hours, are a tremendous addition to and complication of the day's work. Patients little recognise how for the sake of fulfilling a fixed engagement a whole day's work may be deranged. In a moment of happy optimism one makes an appointment for a fixed time some days ahead, but when the time comes it often happens that, so far as money is concerned, it would have been far more profitable to have sat still in one's consulting-room than to have rattled off to a distant suburb even for what may seem a heavy fee. And so with meetings. When the time comes the promise is often by no means easy to fulfil. The day of the meeting of the National Pension Fund for Nurses a letter was received from a distinguished physician who had promised to attend, which serves to illustrate the point we raise, namely, the sacrifice in worry, in money, and in time which may be involved in such an apparently simple thing as attending a public meeting. The letter says: "I had made my arrangements so as to attend to-morrow at four o'clock, and I hope to appear within a few minutes of that hour. I have, however, received late this evening an urgent summons to [naming a distant town], and my return train only reaches St. Pancras at 3.40. I do not as a rule take country journeys till the early afternoon, but I am sacrificing my morning work and my other engagements in order to attend the meeting of the Royal National Pension Fund for Nurses." It looks a little thing to "drop in" at a public meeting and make a few remarks. To busy men, however, it may mean complicated arrangements and no slight amount of money loss. Whether these busy men are wise in running their lives so fine is another question.

### Competing Universities.

Lord Kimberley raised a very important question in the House of Lords last Friday in regard to the affiliation of colleges to the various universities which seem likely to spring up in the large centres of population. The matter arose in reference to a clause in the charter of the Birmingham University, in accordance with which their university is empowered "to admit to affiliation with it or to any of its privileges any college or institution, or the members or students thereof," without any such limitation of the area within which such colleges must be situated, as is prescribed by the new statutes of the University of London, which provided that all institutions admitted to affiliation with it must be situated within the administrative county of London. In the case of Birmingham there was no limitation of the kind, and the result would be that the new university would be able to affiliate to itself any school or institution in any part of the country. The Duke of Devonshire, in replying, referred to the case of the Victoria University, which had the same powers in regard to any institution, if it were satisfied as to the completeness of the curriculum, the sufficiency of the staff, &c. It seems to us, however, quite clear that as the country becomes more thickly planted with universities some territorial limitation should be placed upon them in regard to the area from within which they should affiliate colleges, lest a downgrade competition should be set up among them.

